# Sandensolide Induces Oxidative Stress-Mediated Apoptosis in Oral Cancer Cells and in Zebrafish Xenograft Model

**DOI:** 10.3390/md16100387

**Published:** 2018-10-16

**Authors:** Chung-I Yu, Chung-Yi Chen, Wangta Liu, Po-Chih Chang, Chiung-Wei Huang, Kuang-Fen Han, In-Pin Lin, Mei-Ying Lin, Chien-Hsing Lee

**Affiliations:** 1 Department of Orthopedics, Chi Mei Medical Center, Liouying, Tainan 73659, Taiwan; tlig9212148@gmail.com; 2 Department of Nutrition and Health Science, School of Medical and Health Sciences, Fooyin University, Kaohsiung 83102, Taiwan; xx377@fy.edu.tw; 3 Department of Biotechnology, Kaohsiung Medical University, Kaohsiung 80708, Taiwan; liuwangta@kmu.edu.tw; 4 Division of Thoracic Surgery, Department of Surgery, Kaohsiung Medical University Hospital, Kaohsiung 80708, Taiwan; dr.changpochih@hotmail.com; 5 Weight Management Center, Kaohsiung Medical University Hospital, Kaohsiung 80708, Taiwan; 6 College of Medicine, Kaohsiung Medical University, Kaohsiung 80708, Taiwan; 7 Department of Physiology, Graduate Institute of Medicine, College of Medicine, Kaohsiung Medical University, Kaohsiung 80708, Taiwan; g10054b@ms51.hinet.net; 8 Department of Nursing, Min-Hwei Junior College of Health Care Management, Tainan City 73658, Taiwan; kfhan@mail.mhchcm.edu.tw; 9 Department of Pharmacology, Graduate Institute of Medicine, College of Medicine, Kaohsiung Medical University, Kaohsiung 80708, Taiwan; inpin71126@msn.com; 10 Cancer Center, Kaohsiung Medical University Hospital, Kaohsiung 80708, Taiwan; a0922063039@gmail.com; 11 Department of Medical Research, Kaohsiung Medical University Hospital, Kaohsiung 80708, Taiwan

**Keywords:** human oral squamous cell carcinoma, sandensolide, reactive oxygen species

## Abstract

Presently, natural sources and herbs are being sought for the treatment of human oral squamous cell carcinoma (OSCC) in order to alleviate the side effects of chemotherapy. This study investigates the effect of sandensolide, a cembrane isolated from *Sinularia flexibilis*, to inhibit human OSCC cell growth with the aim of developing a new drug for the treatment of oral cancer. In vitro cultured human OSCC models (Ca9.22, SCC9 and HSC-3 cell lines) and oral normal cells (HGF-1), as well as a zebrafish xenograft model, were used to test the cytotoxicity of sandensolide (MTT assay), as well as to perform cell cycle analysis and Western blotting. Both the in vitro bioassay and the zebrafish xenograft model demonstrated the anti-oral cancer effect of sandensolide. Moreover, sandensolide was able to significantly suppress colony formation and induce apoptosis, as well as cell cycle arrest, in OSCC by regulating multiple key proteins. Induction of reactive oxygen species (ROS) was observed in sandensolide-treated oral cancer cells. However, these apoptotic changes were rescued by NAC pretreatment. These findings contribute to the knowledge of the model of action of sandensolide, which may induce oxidative stress-mediated cell death pathways as a potential agent in oral cancer therapeutics.

## 1. Introduction

The incidence of oral squamous cell carcinoma (OSCC) is increasing globally, particularly in the Asia-Pacific region [1,2]. OSCC is also the main cause of cancer death in males in Taiwan and is ranked there as the fourth leading cause of death overall [3]. The major risk factors result from men’s tobacco use, drinking alcohol, and chewing betel nut [3]. The main treatment strategies for OSCC include surgery, radiation therapy, chemotherapy and targeted therapy. However, intrinsic and acquired drug resistance becomes a major obstacle for OSCC therapy, resulting in limited chemotherapy treatments. Therefore, there is an urgent need to develop effective strategies for OSCC treatment.

Several reports have documented the role of reactive oxygen species (ROS) in a number of pathophysiological states including cancer. At the very early stage of cancer, ROS attack DNA and additional cellular components such as lipids, proteins, leaving behind reactive species that can couple to DNA bases [4]. The accumulation of DNA damage resulting from incomplete repair or disrepair may lead to DNA lesion, mutagenesis and consequently cancerous transform generation [5]. Several natural compounds targeting ROS has been reported to regulate apoptosis for selective killing in oral cancer [6,7,8,9]. Thus, the reduction of ROS might play a critical role in regulating the selective activation of apoptosis for selective killing in oral cancer chemotherapy.

Marine microbes have potential as upcoming and promising sources for the development of anticancer drugs [10,11,12,13]. Recently, many soft coral-derived compounds have also been reported as having potential applications as anticancer drugs. For example, *Sinularia* lochmodes-derived sinuleptolide possess anti-inflammatory and anticancer properties [13,14,15]. The cytotoxic effects of 24-methyl-cholesta-5, 24(28)-diene-3β,19-diol-7β-monoacetate (MeCDDA), isolated from soft coral (*Nephthea erecta*), has been found to be cytotoxic against different types of human cancer cell lines [16]. 3β, 11-dihydroxy-9,11-secogorgost-5-en-9-one from soft coral induces apoptosis and autophagy in breast cancer cells [17]. Sandensolide was isolated from the soft coral *Sinularia flexibilis* [18]. However, no studies have investigated the effects of sandensolide in the treatment of cancer. The present study investigated the growth inhibitory effects and underlying mechanism of action of sandensolide in human OSCC in a series of models in vitro and in vivo.

## 2. Results

### 2.1. Effect of Sandensolide on the Cell Viability of Oral Cancer Cells

**Figure 1:**
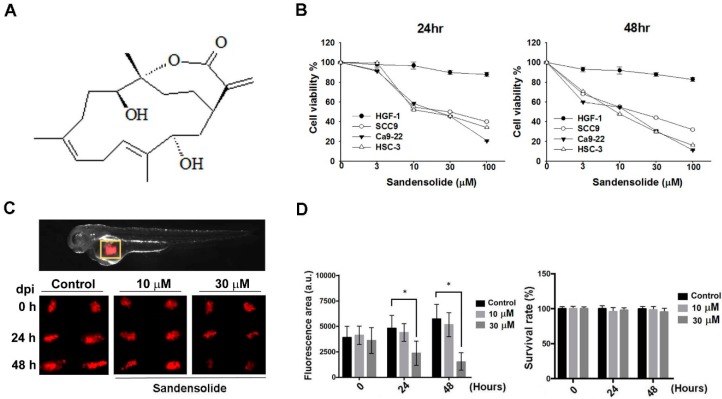
Effect of sandensolide on the proliferation of OSCC cells. (**A**) Structure of sandensolide. (**B**) Three OSCC models (SCC9, Ca9.22 and HSC-3 cells) and oral normal cells (HGF-1) were treated with various concentrations of sandensolide for 24 h and 48 h, respectively. Cell growth of the vehicle-treated group is set as 100%. (**C**) The tumor volume in the zebrafish xenograft model. The intensity of red fluorescence is proportional to the xenograft tumor size. N = 20 embryos for each group. (**D**) The quantitative analysis of C in the left part. The right figure shows the survival rate of the zebrafish xenograft model after indicated treatment. Values are expressed as means ± S.D. (n ≥ 4, * *p* < 0.05 relative to the vehicle-treated control group).

**Figure 2:**
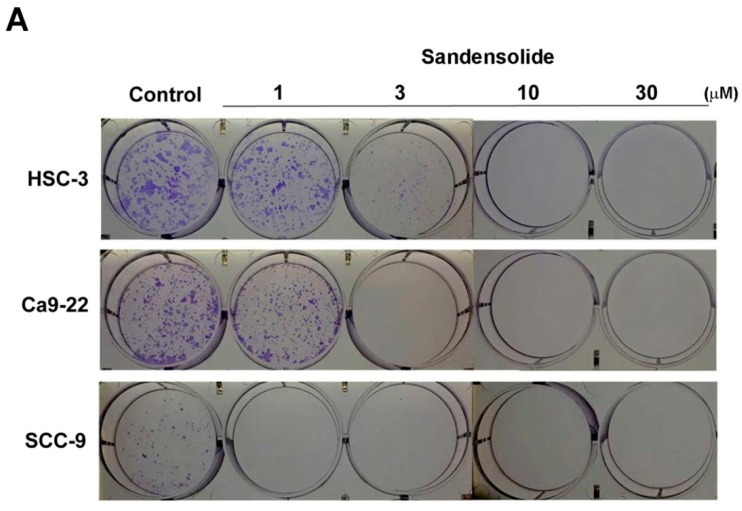

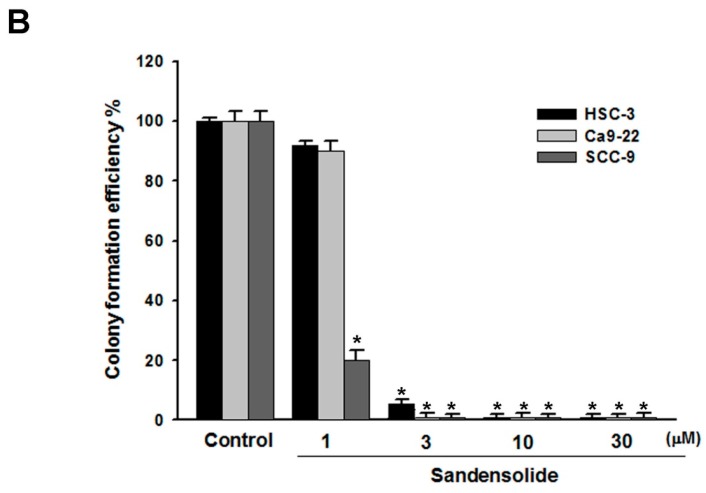
Effect of sandensolide on clonogenic ability of OSCC cells. (**A**) Three OSCC models (SCC9, Ca9.22 and HSC-3 cells) were seeded at a density of 100 cells per well in 6 well plates. After 14 days of growth, the cells were stained with crystal violet and the stained plates were scanned. Representative wells are shown. (**B**) Crystal violet stained colonies were quantified. Values are expressed as means ± S.D. (n ≥ 4, * *p* < 0.001 relative to the vehicle-treated control group).

To examine the growth-inhibitory effect of sandensolide (Figure 1A) in human OSCC models (SCC9, Ca9.22 and HSC-3 cell lines) and oral normal cells (HGF-1), we first treated them with various concentrations of sandensolide for 24 h and 48 h, assessed by MTT assay. As shown in Figure 1B, significant inhibition of proliferation was shown at 3, 10, 30 and 100 μM sandensolide in both dose- and time-dependent manners, but no toxicity was observed in oral normal (HGF-1) cells. The EC50 of sandensolide at 48 h on SCC9, Ca9.22 and HSC-3 cells was, respectively, 30.21, 20.17 and 13.57 μM. In addition, we also evaluated the antitumor efficacy of sandensolide in vivo. HSC-3 cells were implanted into the yolk sac of zebrafish larvae followed by incubating with different sandensolide concentrations for the indicated times. We found that the observed tumor sizes, as indicated by the intensity of red fluorescence, were reduced exposed to 30 μM sandensolide without obvious survival rate alteration (Figure 1C,D). To assess the long-term inhibitory effect of sandensolide on the transforming properties of OSCC cells, we performed a colony formation assay. Sandensolide significantly reduced the number of colonies compared with the control group (*p* < 0.001; Figure 2A) and in a dose-dependent manner (Figure 2B). These results indicate the anti-cancer potential of sandensolide on OSCC cells.

### 2.2. Effect of Sandensolide on Cell Cycle Arrest of Oral Cancers

**Figure 3:**
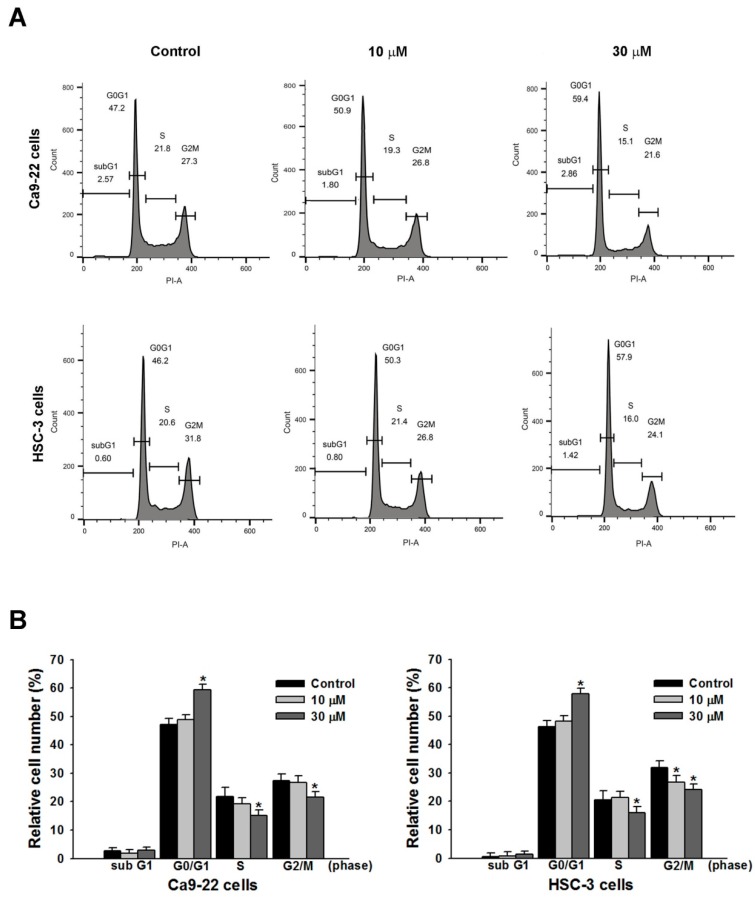
Modulation of sandensolide on cell cycle in OSCC cells. (**A**) Cells were treated with 10 or 30 μM sandensolide, as indicated, for 24 h. The cell cycle distribution was analyzed through flow cytometry with PI staining. (**B**) Cell cycle data for A. Values are expressed as means ± S.D. (n ≥ 3, * *p* < 0.05 compared to the vehicle-treated control group).

To elucidate the mechanism of growth inhibition on OSCC cells, the effects of sandensolide on cell cycle progression were determined in Ca9.22 and HSC-3 cells. Figure 3 shows that sandensolide caused significant changes in the cell cycle distribution of Ca9.22 and HSC-3 cells. After incubation with 30 μM sandensolide, the proportion of G0/G1 phase cells reached 60.10 ± 1.26% and 58.60 ± 1.25% in Ca9.22 and HSC-3 cells, respectively, as compared to the control groups (47.1 ± 0.80% and 45.20 ± 0.36% in Ca9.22 and HSC-3 cells, respectively), suggesting that sandensolide caused G0/G1 phase arrest in OSCC cells.

**Figure 4:**
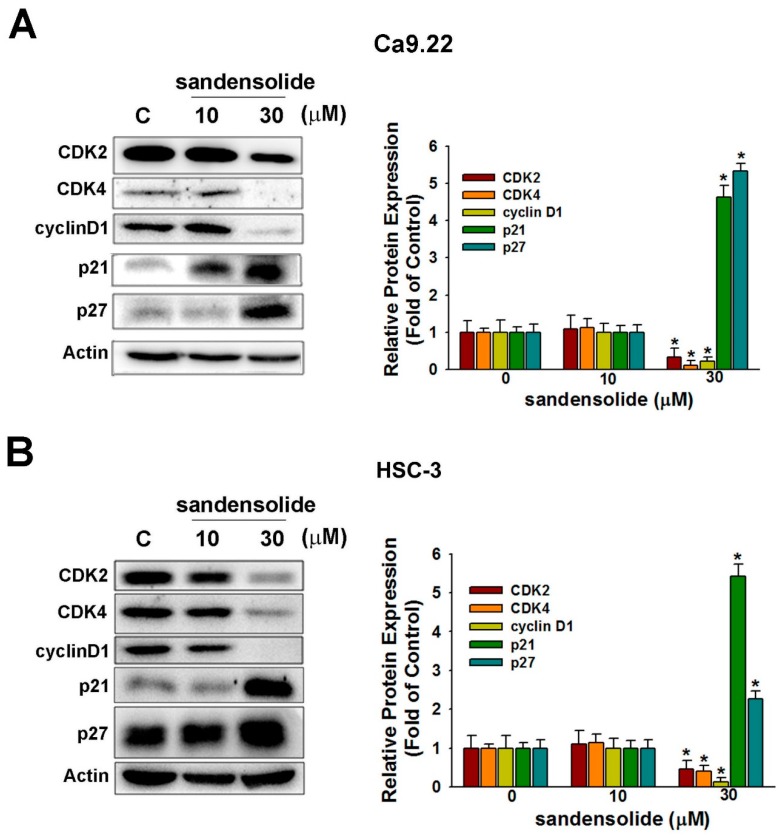
Influence of sandensolide on cell cycle-related proteins in OSCC cells. In Ca9.22 (**A**) and HSC-3 (**B**) cells treated with sandensolide. The protein level of CDK2, CDK4, cyclin D1, p21 and p27 was determined by immunoblotting assays. The fold-induction data are expressed as the intensity of the protein bands produced from the target proteins/β-actin relative to that of the vehicle-treated group. Values are expressed as means ± S.D. (n ≥ 5, * *p* < 0.05 compared to the vehicle-treated control group).

Consistently, with application of sandensolide, the cell cycle regulatory proteins (cyclin-dependent kinase; CDK2, CDK4 and cyclin D1) decreased, whereas cyclin-dependent kinase inhibitors (p21 and p27) increased (Figure 4A). The results of the quantitative analysis are presented in Figure 4B, indicating that sandensolide inhibits OSCC cells growth through arresting the cell cycle at the G0/G1 phase by modulating cell cycle regulatory proteins and cyclin-dependent kinase inhibitors.

### 2.3. Effect of Sandensolide on Apoptosis of Oral Cancers

**Figure 5:**
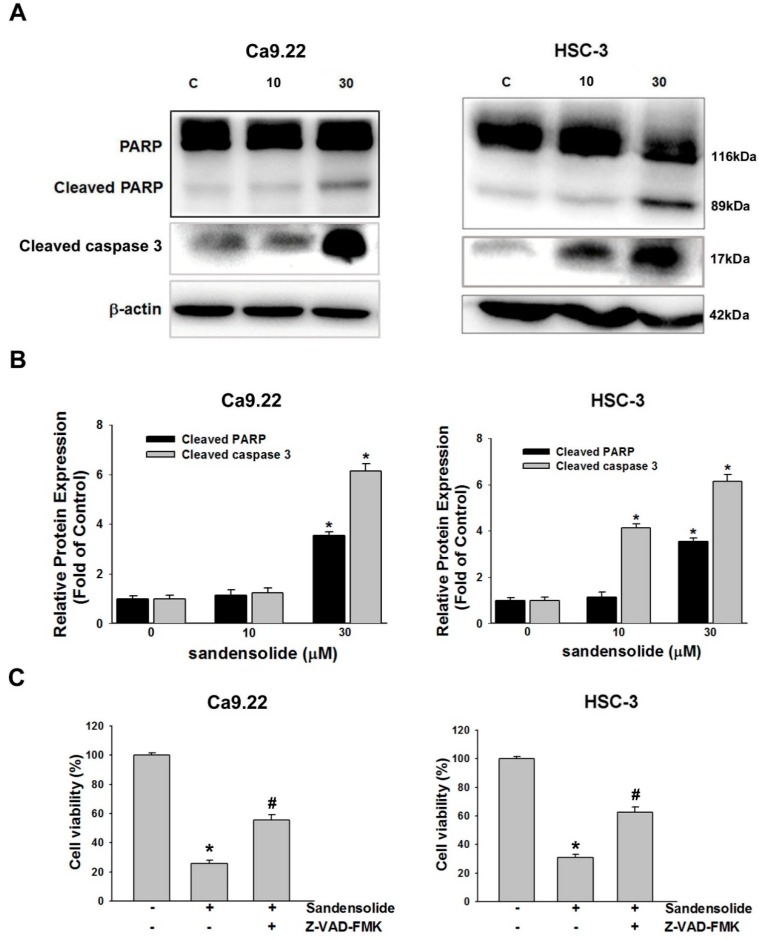
Regulation of sandensolide on apoptosis in OSCC cells. (**A**) OSCC (Ca9.22 and HSC-3) cells were treated with sandensolide (10 and 30 μM) for 24 h. Protein samples were subjected to western blot analysis with antibodies against cleaved caspase-3/caspase-9, cleaved PARP and β-actin, and relative band densities were analyzed. (**B**) The fold-induction data are expressed as the intensity of the protein bands produced from the target proteins/β-actin relative to that of the vehicle-treated group. (**C**) Percentage cell viability assessed by MTT assay in OSCC (Ca9.22 and HSC-3) cells exposed to sandensolide with or without 10 μM Z-VAD-FMK (a pan-caspase inhibitor). Data are presented as bar graphs. Values are expressed as means ± S.D. (n ≥ 5, * *p* < 0.05 relative to the vehicle-treated control group, ^#^ *p* < 0.05 compared to sandensolide-treated cells).

To investigate the underlying mechanisms by which sandensolide induces apoptosis in OSCC cells, we determined the effect of sandensolide on caspases signaling. Western blotting revealed that sandensolide significantly increased the cleavage of effector caspases such as caspase-3 and poly (ADP-ribose) polymerase (PARP), the substrate of caspases-3 (Figure 5A,B). The results demonstrated that sandensolide-induced apoptosis in OSCC cells by activating caspase-3 and cleaving PARP. Moreover, the sandensolide-mediated cell viability of OSCC cells was abolished by pre-treatment of a pan-caspase inhibitor, 10 μM Z-VAD-FMK (Figure 5C), suggesting that sandensolide-induced apoptosis of OSCC cells occurs through caspase-dependent pathway. Taken together, the results demonstrated that sandensolide induces apoptosis via activation of caspases in human OSCC cells.

### 2.4. Effect of Sandensolide on ROS generation of Oral Cancers

**Figure 6:**
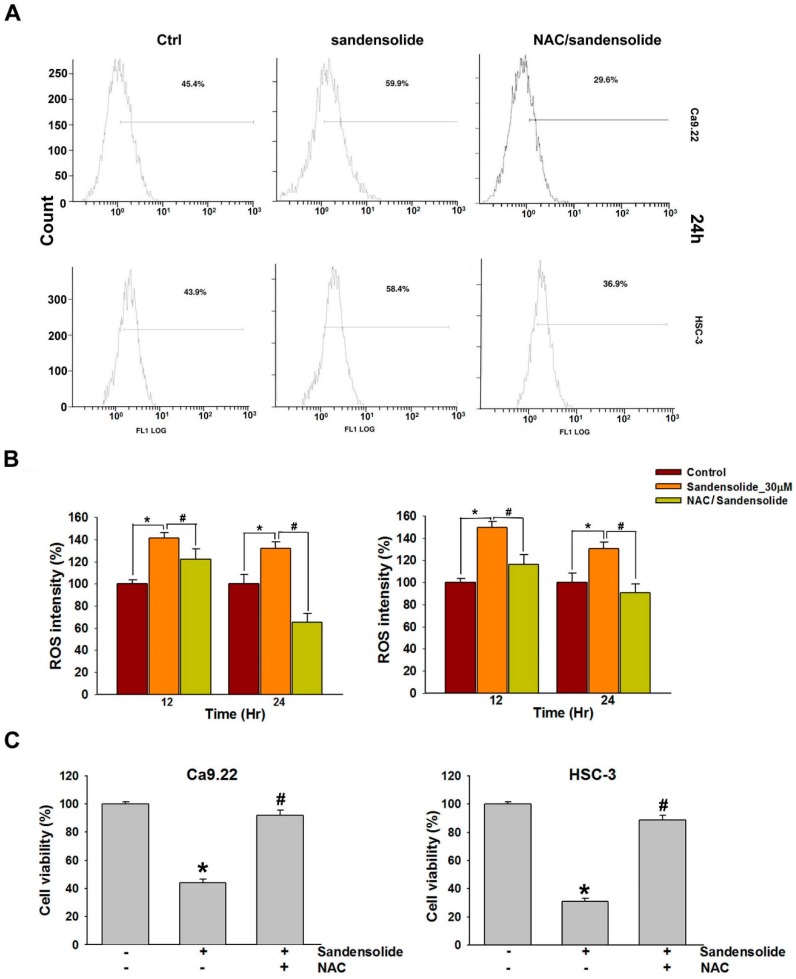
ROS levels of sandensolide-treated OSCC cells and changes after pretreatment with NAC. (**A**) Representative flow cytometry-based ROS patterns of sandensolide-treated OSCC (Ca9.22 and HSC-3) cells in the presence or absence of NAC (an inhibitor of ROS). (**B**) Quantification of ROS intensity in (**A**). (**C**) Percentage cell viability assessed by MTT assay in OSCC (Ca9.22 and HSC-3) cells exposed to sandensolide in the presence or absence of 5 mM NAC. Values are expressed as means ± S.D. (n ≥ 3, * *p* < 0.05 relative to the vehicle-treated control group, ^#^ *p* < 0.05 compared to sandensolide-treated cells).

Figure 6A shows the ROS flow cytometry patterns of Ca9.22 and HSC-3 cells after the addition of sandensolide for 12 h. The relative ROS-positive staining of sandensolide (30 μM)-treated Ca9.22 and HSC-3 cells increased over time (Figure 6B). ROS flow cytometry shown the patterns of N-acetyl-L-cysteine (NAC) effects against sandensolide-induced ROS generation (Figure 6A,B). Furthermore, to confirm the role of ROS in sandensolide-induced anti-proliferative effects, we treated cells with sandensolide in presence and absence of 5 mM antioxidant, NAC. The cytotoxicity effects of sandensolide-induced OSCC cells were reversed by NAC (Figure 6C). These results suggest that ROS production is essential for the anti-proliferative activity of sandensolide in OSCC cells.

### 2.5. Effect of Sandensolide on ROS-Mediated Caspases-Based Apoptosis of Oral Cancers

**Figure 7:**
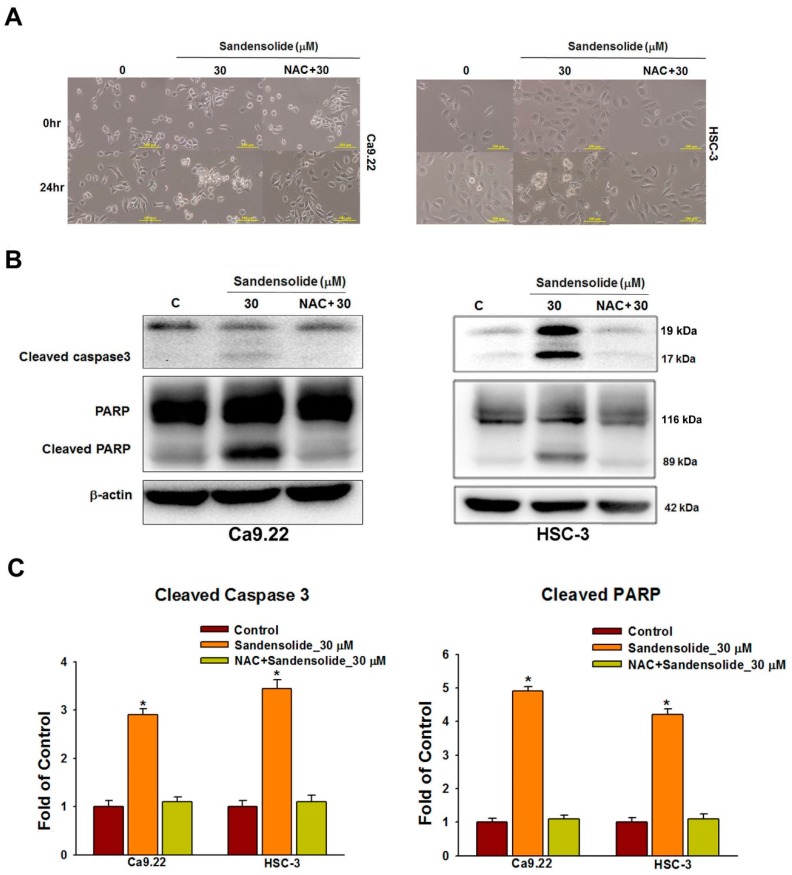
Caspase-based apoptosis in sandensolide-treated OSCC cells with or without pretreatment with NAC. (**A**) Cell morphology changes in sandensolide-treated OSCC (Ca9.22 and HSC-3) cells for 24 h with or without pretreatment with NAC. Cell images were captured at 20x magnification. (**B**) Expression of the apoptosis-related proteins (caspase 3 and cleaved PARP) in sandensolide-treated OSCC cells was analyzed by western blotting. β-actin was used as an internal control. (**C**) The fold-induction data are expressed as the intensity of the protein bands produced from the target proteins/β-actin relative to that of the vehicle-treated group. Data are presented as a bar graph. Values are expressed as means ± S.D. (n ≥ 5, * *p* < 0.05 compared to the vehicle-treated control group).

Figure 7A shows the cell morphology change of sandensolide-treated OSCC cells over time. After 24 treatments of sandensolide, the morphological features of apoptosis, such as apoptotic bodies and cell shrinkage, were visualized. In contrast, NAC pretreatment was able to prevent these apoptotic morphologies. To examine the involvement of specific apoptosis signaling proteins in sandensolide-induced ROS generation on OSCC cells, the time course change of apoptosis signaling proteins, PARP and cleaved caspase 3 was performed. As shown in Figure 7B, cleaved PARP and caspase 3 increased at 24 h application of sandensolide in OSCC cells. The results of the quantitative analysis are presented in Figure 7C, suggesting that sandensolide-induced death of OSCC cells involved with ROS.

## 3. Discussion

The application of natural products for chemoprevention and therapy has been becoming a more and more important issue over the past three decades [19,20,21]. Several studies have shown that some marine-derived compounds possess biological activity and pharmacological effect in cancer models with little or no side effects [22,23,24]. To the best of our knowledge, this is the first study illustrating the anti-cancer effect of sandensolide on OSCC cells and a zebrafish xenograft model, which also highlights the possible mechanism underlying the cytotoxic effect of sandensolide. Our study clearly demonstrated that sandensolide-mediated ROS generation alters the expression of proteins involved in colony formation, cell cycle and cell survival, thereby regulating growth inhibition and apoptosis.

Marine organisms are the cradle for many excellent pharmaceutical products, particularly soft corals [25,26,27]. Several types of cembranoid compounds with prominent biological activities have been isolated from *Sinularia flexibilis* [28,29,30,31,32]. Numerous marine metabolites in Taiwanese soft corals have also been found from *Sinularia flexibilis*, with anti-inflammatory activity [31,33], neuroprotective effect [34] and cytotoxic activity against various cancer cells lines [35,36,37]. Furthermore, sinulariolide is an active natural product isolated from *Sinularia flexibilis* and was found to display notable anti-cancer activity including on bladder cancer, hepatocellular carcinoma and melanoma cells [37,38,39]. Another natural product isolated from *Sinularia flexibilis*, sinularin, has been shown to possess antineoplastic activity against human hepatocellular carcinoma [40] and gastric carcinoma [36]. Sandensolide isolated from the soft coral *Sinularia flexibilis* [18] is the first compound discovered there to possess cytotoxicity against OSCC cells.

Oxidative stress was correlated with OSCC, as the reduction of ROS was observed in patients suffering from advanced oral cancer; however, high ROS levels induce OSCC cell death [41]. Therefore, ROS is an important anticancer target in OSCC therapeutic strategies. A large number of studies on various medicinal herbs have shown that they may induce ROS-mediated apoptosis in OSCC cells. For example, erufosine is able to induce ROS in OSCC cell lines and the loss of mitochondrial membrane potential [42]. β-lapachone produces reactive oxygen species-mediated apoptosis in human OSCC cells [6]. In addition, recent studies demonstrated that soft coral compounds mediate ROS induced apoptosis and DNA damage in OSCC cells in vitro [7,13]. In the present study, we found that sandensolide-induced ROS generation and apoptosis were abolished by the antioxidant NAC, indicating that ROS is a critical mediator involved in sandensolide-induced apoptosis in OSCC.

In this study, we found that the cytotoxic effect of sandensolide on OSCC cells partially reversed by NAC and Z-VAD-FMK, suggesting that other pathways may be involved in the activity of this compound. Several studies have demonstrated that the accumulation of ROS induces oxidative damage to the induction of autophagy, leading to subsequent production of apoptotic cell death [43,44]. The cytotoxic effect of sandensolide related to autophagy should be an interesting subject for further investigations.

In conclusion, we demonstrate that the anticancer potential of sandensolide in OSCC cells in vitro and in vivo. This cell-killing mechanism includes ROS generation and apoptosis, which can be rescued by NAC pretreatment. Therefore, these results suggest that sandensolide has an anticancer potential for oxidative stress-mediated oral cancer therapy based on the cell line study.

## 4. Materials and Methods

### 4.1. Chemicals and Reagents

The marine natural compound, sandensolide, was isolated from the soft coral *S. flexibilis* as described [18] and kindly provided by Prof. Chung-Yi Chen (National Museum of Marine Biology & Aquarium, Pingtung, Taiwan). The following compounds were obtained from Gibco BRL (Gaithersburg, MD, USA): DMEM medium, fetal bovine serum (FBS), trypan blue, penicillin G, and streptomycin. Dimethyl sulphoxide (DMSO), CPT, ribonuclease A (RNase A), acetic acid, methanol, N-acetylcystein (NAC) and 3-[4,5-Dimethylthiazol-2-yl]-2,5-diphenyltetrazolium bromide (MTT) were purchased from Sigma-Aldrich. Propidium iodide (PI) was purchased from BD Biosciences. Antibodies against XIAP and β-actin were obtained from Santa Cruz Biotechnology (Santa Cruz, CA, USA). Antibodies against cleaved caspase-3 and PAPP were purchased from Cell Signal Technology (San Jose, CA, USA). Anti-mouse and anti-rabbit IgG peroxidase-conjugated secondary antibodies were purchased from Pierce (Rockford, IL, USA). The anti-rabbit Rhodamine-conjugated antibody was purchased from Abcam (Cambridge, UK).

### 4.2. Cell Lines and Culture

Human oral cancer cell lines from gingival carcinoma (Ca9.22) and tongue carcinoma (SCC9 and HSC-3) were respectively ordered from Health Science Research Resources Bank (HSRRB) (Osaka, Japan) and American Type Culture Collection (ATCC; Manassas, VA, USA). A normal human gingival fibroblast cell line (HGF-1) was ordered from ATCC. All tested cells were maintained in DMEM: F-12/3:2 ratio and supplemented with 10% FBS, 2 mM glutamine, and antibiotics (100 units/mL penicillin and 100 μg/mL streptomycin) at 37 °C in a humidified atmosphere of 5% CO_2_.

### 4.3. Cell Viability

Determination of live cell numbers is often used to assess the rate of cell proliferation caused by drugs and cytotoxic agents. MTT is a yellow tetrazolium salt that may enter cells, and succinic dehydrogenase enzyme in live cells oxidizes MTT to yield a water-insoluble purple formazan crystal. This study used MTT assays to examine the cell survival and human OSCC models (SCC9, Ca9.22 and HSC-3 cell lines) and oral normal cells (HGF-1) after sandensolide treatment. Briefly, cells at 104 cells/mL were seeded onto 96-well plates (100 μL/well) and incubated with different concentrations of sandensolide (1, 3, 10, 30 and 100 μM) for 24 and 48 h. After adding 100 μL MTT solutions (1 mg/mL in PBS) to each well, the culture was incubated at 37 °C for 4 h, following which 100 μL DMSO were added to dissolve the formazan. The plate was read on an ELISA microplate reader (EZ Read 400 Research, BioChrom, Holliston, MA, USA) at an absorbance of 595 nm.

### 4.4. Colony Formation Assay

For the colony formation assays, the human OSCC models (SCC9, Ca9.22 and HSC-3 cell lines) were seeded into 6-well plates (Corning Incorporated) at a density of 1 ×  10^2^ cells per well. Subsequently, the cells were treated with 0, 1, 3, 10, 15 or 30 µM sandensolide and incubated for 14 days in a humidified atmosphere of 5% CO_2_ at 37 °C. Subsequently, the cells were fixed with colony fixation solution (acetic acid/methanol 1:7 (vol/vol)) for 10 min at room temperature and stained with crystal violet for 15 min at room temperature, followed with colony counting by eye. Images were captured using a fluorescence microscope (Eclipse TS100; magnification, ×10; Nikon Corporation, Tokyo, Japan).

### 4.5. Cell Cycle Analysis

5  × 10^5^ OSCC cells were seeded onto 10 cm petri dishes and treated with or without sandensolide for 12 h. Subsequently, cells were harvested and stained with PI staining kit according to the manufacturer’s manual. Cells were analyzed by flow cytometry (FACS Calibur; Becton Dickinson, Mountain View, CA, USA) using WinMDI 2.9 software (written by Joseph Trotter, Scripps Research Institute, La Jolla, CA, USA).

### 4.6. Intracellular ROS Determination

The ROS reacting dye 2′,7′-dichlorodihydrofluorescein diacetate (DCFH-DA) was chosen. Cells were seeded at a density of 5 × 10^5^ cells/mL medium per well of 6-well plates for overnight growth. After sandensolide treatment for 12 or 24 h, cells were reacted with 100 nM H2DCF-DA in phosphate-buffered saline (PBS) for 30 min at 37 °C. After harvesting and washing, cells were resuspended in PBS for ROS detection using an Accuri C6 flow cytometer (Becton-Dickinson, Mansfield, MA, USA) and its built-in software.

### 4.7. Western Blot Analysis

Western blotting was carried out as described previously [45]. Briefly, cells were harvested and lysed. Lysates were centrifuged, and the protein concentration was determined. Equal amounts of protein were separated by SDS-polyacrylamide gel electrophoresis (SDS-PAGE) and then electrotransferred. The membrane was blocked with 5% non-fat milk, followed by incubation with primary and secondary antibodies against specific proteins. The signals were detected using enhanced chemiluminescence (ECL) detection kit (Amersham Piscataway, NJ, USA).

### 4.8. Zebrafish Xenograft Assay

The zebrafish (Danio rerio) Tg (fli1:EGFP) were obtained from Taiwan Zebrafish Core Facility at Academia Sinica (TZCAS, Taipei, Taiwan). The care and maintenance of zebrafish were handled in compliance with the animal care regulations and standard protocols of the animal center (Kaohsiung Medical University Hospital, Kaohsiung, Taiwan) for zebrafish adults and larvae). Zebrafish were kept at 28.5 °C in aquaria with day/night light cycles (10 h dark vs. 14 h light periods).

### 4.9. Zebrafish Xenograft Assay

The zebrafish xenograft assay was used for confirming the inhibitory effect of sandensolide on OSCC cells. The use of zebrafish complied with the principles of 3Rs (Reduction, Replacement and Refinement), and the approval protocol by Institutional Animal Care and Use Committee (IACUC) of Kaohsiung Medical University Hospital, Kaohsiung, Taiwan (IACUC Approval No. KMU-IACUC-107064). Human oral cancer cells (HSC-3) were labeled with DiI dye (Molecular Probes, Carlsbad, CA, USA) and injected into zebrafish in order to track the cells using fluorescence microscopy. The procedure was performed according to a previous study with minor modifications [46,47]. Briefly, 48 h post-fertilization (hpf) zebrafish embryos were anesthetized with 0.01% of tricaine and transplanted with about 50 HSC-3 cells per embryo. After confirmation of the localized DiI-labeled cell mass at the injection site, the zebrafish were incubated in water at indicated concentrations of sandensolide for 24 and 48 h post-injection (hpi), respectively. The cancer cell proliferation was determined by visualizing dissemination of DiI-labeled cell from the injection site using an inverted microscope (Nikon Eclipse TE2000-U, Tokyo, Japan).

### 4.10. Statistical Analysis

Differences between sandensolide- and DMSO- (as vehicle control) treated cells were analyzed in at least triplicate experiments. The significance of the differences was analyzed by one-way analysis of variance (ANOVA) with Tukey’s post-hoc test, with *p* < 0.05 considered significant.
